# Genome-Wide Analysis of Odorant and Gustatory Receptors in Six *Papilio* Butterflies (Lepidoptera: Papilionidae)

**DOI:** 10.3390/insects13090779

**Published:** 2022-08-29

**Authors:** Ningna Yin, Haiyan Xiao, Anjin Yang, Chun Wu, Naiyong Liu

**Affiliations:** Key Laboratory of Forest Disaster Warning and Control of Yunnan Province, Southwest Forestry University, Kunming 650224, China

**Keywords:** *Papilio*, odorant receptor, gustatory receptor, gene structure, olfaction, gustation, reproduction

## Abstract

**Simple Summary:**

The butterflies of the Lepidoptera are representatives of herbivorous insects. Host plant diversities may shape the evolution of chemosensory receptors in butterflies. However, little is known about whether the host plant range is correlated with the size differences of chemoreceptors as key molecular targets for butterfly–plant chemical interactions. Here, we characterized 381 odorant (ORs) and 328 gustatory (GRs) receptors in one *Papilio* generalist and five other *Papilio* specialists regarding their comprehensive annotation, gene structure, phylogenetics and expression profiles. Among six *Papilio* species, orthologous ORs or GRs shared conserved gene structure, but the differences in intron size frequencies were observed between the generalist and the specialists. Furthermore, expression profiles identified candidate OR and GR genes in antennae, foretarsi or reproductive tissues of *P. xuthus*. This study addresses, for the first time, the issue that the breath of host plants does not appear to result in the obvious expansions of ORs and GRs in *Papilio* butterflies and identifies candidate molecular targets associated with olfaction, oviposition or reproduction in *P. xuthus*.

**Abstract:**

The chemical interactions of insects and host plants are shaping the evolution of chemosensory receptor gene families. However, the correlation between host range and chemoreceptor gene repertoire sizes is still elusive in Papilionidae. Here, we addressed the issue of whether host plant diversities are correlated with the expansions of odorant (ORs) or gustatory (GRs) receptors in six *Papilio* butterflies. By combining genomics, transcriptomics and bioinformatics approaches, 381 ORs and 328 GRs were annotated in the genomes of a generalist *P. glaucus* and five specialists, *P. xuthus*, *P. polytes*, *P. memnon*, *P. machaon* and *P. dardanus*. Orthologous ORs or GRs in *Papilio* had highly conserved gene structure. Five *Papilio* specialists exhibited a similar frequency of intron lengths for ORs or GRs, but which was different from those in the generalist. Phylogenetic analysis revealed 60 orthologous OR groups, 45 of which shared one-to-one relationships. Such a single gene in each butterfly also occurred in 26 GR groups. Intriguingly, bitter GRs had fewer introns than other GRs and clustered into a large clade. Focusing on the two chemoreceptor gene families in *P. xuthus*, most PxutORs (52/58) were expressed in antennae and 31 genes in reproductive tissues. Eleven out of 28 foretarsus-expressed PxutGRs were female-biased genes, as strong candidates for sensing oviposition stimulants. These results indicate that the host range may not shape the large-scale expansions of ORs and GRs in *Papilio* butterflies and identify important molecular targets involved in olfaction, oviposition or reproduction in *P. xuthus*.

## 1. Introduction

The swallowtail butterfly genus *Papilio* is constituted of a comparable number of members in the family Papilionidae, with over 200 described species [1,2]. As the majority of *Papilio* species are specialized herbivores with their larvae feeding solely on the plants in a family or a genus, they are typical oligophagous insects, such as *P. xuthus*, *P. polytes*, *P. memnon*, *P. machaon* and *P. dardanus* [3,4]. Exceptionally, the eastern tiger swallowtail butterfly, *P. glaucus*, uses a broader range of plants from at least 14 families as its feeding and ovipositing hosts, facilitating the study of the size differences of chemosensory gene families between generalists and specialists in Papilionidae [5,6]. For the oligophagous butterflies, although they feed exclusively on a limited number of plants, multiple factors, including different habitats, feeding hosts, ecological niches and human activities, are broadening and changing their host range. Such a result possibly contributes to the increase in specific gene families in relation to chemoreception. Hence, the host plant range is regarded as an important driving force for the expansions of chemosensory-associated gene families in herbivorous insects, particularly for odorant receptors (ORs) and gustatory receptors (GRs) mainly involved in smell and taste, respectively [7,8,9,10,11,12,13].

Insect OR is one representative of the most important olfactory-sensing gene families responsible for the detection of semiochemicals produced by conspecifics (e.g., sex pheromones) and host/non-host plants (e.g., volatile compounds) [14,15]. In moths, females, in general, release sex pheromones to attract males, of which male antenna-biased ORs (called pheromone receptors, PRs) are key molecular targets for detecting the components. The PRs comprise a highly conserved PR clade and a recently proposed novel PR clade, with the latter ranging from 1 to 13 genes. However, most members of this novel PR clade are yet to be functioned [16,17,18,19]. Outside PR members, the remaining ORs, but not including an olfactory receptor coreceptor (Orco), are able to perceive a variety of host plant volatiles. Thus, the breadth of host plants possibly determines the repertoire sizes of olfactory-related genes such as ORs, as implied in Coleoptera [7,20]. In other insect Orders, OR numbers are not correlated with the host range. For example, a polyphagous herbivore *Bemisia tabaci* (9 to 13 genes) had fewer ORs compared to a monophagous species *Nilaparvata lugens* (141 genes) and an oligophagous species *Laodelphax striatellus* (37 genes) [21,22]. A similar case also occurred in moths of the Lepidoptera, ranging from 72 ORs in *Bombyx mori* (monophagous) and 73 ORs in *Manduca sexta* (oligophagous) to 69, 73 and 84 ORs separately in *Spodoptera frugiperda*, *Spodoptera litura* and *Helicoverpa armigera* (polyphagous) [23,24,25,26,27].

When larvae and adults finish the search and orientation of potential host plants, they need to further assess the quality of hosts. For contact chemoreception, GRs are required for the recognition of non-volatile compounds (i.e., tastants) [28,29,30]. Outside taste receptors sensitive to carbon dioxide (CO_2_), other GRs primarily detect phagostimulants that promote female oviposition and larval feeding behaviors, such as sugars, amino acids, feeding and oviposition stimulants [31,32]. In several moth species, GRs belonging to the sugar or GR43a-like receptor subfamilies were responsible for the perception of various sugars, including D-fructose and non-fructose sugars [33,34,35,36]. In *P. xuthus* and *Pieris rapae*, a GR43a-like PxutGR1 and a bitter PrapGR28 responded separately to synephrine and sinigrin, stimulating larval feeding and adult oviposition [29,37]. On the other hand, other GRs are capable of recognizing phagodeterrents that regulate larval feeding and female oviposition, including bitter compounds, feeding and oviposition deterrents. In *B. mori*, *Plutella xylostella* and *H. armigera*, members of bitter receptors showed responses to feeding or oviposition deterrents and even amino acids [30,38,39]. As indicated in Noctuidae, the diversity of host plants is shaping the evolution of GRs, representing the large-scale expansions of bitter receptor repertoires [23,24,39,40]. In four nymphalid butterflies, the generalist *Vanessa cardui* possessed more GRs compared to those in two specialists *Vanessa indica* and *Polygonia c-aureum*, but almost equal to the GR number in another specialist *Araschnia burejana*. However, GR numbers in these four species were derived exclusively from the transcriptomes of female legs [10]. Thus, it is ambiguous whether the GR repertoire sizes of butterflies are correlated with the host range.

With the emergence of the first genome of *P. glaucus* in the family Papilionidae [41], genomic sequences from other papilionid species were published in succession, up to 20 species in the National Center of Biotechnology Information (NCBI) Genome database (https://ncbi.nlm.nih.gov/genome/, accessed on 29 May 2022), emphasizing their evolutionary biology, mimicry, genetics and speciation. However, information on the relationships between the host plant range and chemosensory gene repertoire sizes in *Papilio* species is still scarce. Here, we assumed that diverse host plants might drive the expansions of ORs and GRs in *Papilio* butterflies associated with host orientation and selection. By combining genomics and bioinformatics strategies, our extensive manual annotation and comparative analyses provide insights into gene gains and losses of ORs and GRs in six *Papilio* species, revealing the diversity and differences of host plants as well as the constantly changeable habitats now possibly do not result in the large-scale expansions of olfactory or gustatory receptors in the genus *Papilio*. The expression profile data of the two chemoreceptors in *P. xuthus* identify candidate molecular targets involved in olfaction, gustation or reproduction.

## 2. Materials and Methods

### 2.1. Bioinformatics

#### 2.1.1. Genome Resource

In order to address the issue of whether the repertoire sizes of OR and GR gene families in *Papilio* butterflies are shaped by the host range, we selected a generalist (*P. glaucus*: host plants from over 14 families) and five specialists (*P. xuthus*, *P. polytes*, *P. memnon*, *P. machaon* and *P. dardanus*: host plants from a family). In order to comprehensively identify and annotate OR and GR genes in six *Papilio* species, we downloaded their genome assemblies from the NCBI Genome database (https://www.ncbi.nlm.nih.gov/genome/, assessed on 10 May 2021). The detailed information on genome versions and accession numbers is as follows: *P. glaucus* (pgl_assembly_v1, GCA_000931545.1) [41], *P. xuthus* (two versions: Pxut_1.0, GCA_000836235.1 and Pap_xu_1.0, GCA_001298345.1) [42,43], *P. polytes* (Ppol_1.0, GCA_000836215.1) [43], *P. memnon* (Pmem_mimetic_representative_assembly_1.0, GCA_003118415.2) [44], *P. machaon* (Pap_ma_1.0, GCA_001298355.1) [45] and *P. dardanus* (ASM1318645v1, GCA_013186455.1) [46]. Further, the corresponding transcriptomes, when necessary, were retrieved from the NCBI Sequence Read Archive (SRA) database (https://www.ncbi.nlm.nih.gov/sra/, assessed on 10 May 2021).

#### 2.1.2. Gene Annotation

A homology-based TBLASTN search was performed against the local genomes, using the protein sequences of ORs and GRs from *B. mori*, *H. armigera*, *M. sexta*, *Danaus plexippus* and *Heliconius melpomene* as queries [11,25,26,27,39,47,48,49]. Considering the low identities of some ORs or GRs among different species, we set an *E*-value of 0.05 and a maximum hit of 40. If one gene was subjected to gaps in the middle of an ORF or incompleteness of 3′– or 5′– termini, the corresponding transcriptomes were used to improve its ORF as soon as possible. In addition, a manual effort was required to determine the boundaries of introns and exons by GeneWise, with a “GT–AG” rule [50]. Further, using identified *Papilio* ORs and GRs as queries, an iterative search strategy was conducted against the genomes until no novel genes were found. In order to check the superfamily (the 7tm_6 superfamily) and conserved domains of ORs and GRs, all identified genes were blasted against the NCBI non-redundant protein sequence database. In order to avoid the identification of repetitive ORs or GRs, only one gene was retained when two or more amino acid sequences shared over 95% identities. If one gene encoded a short sequence and was subjected to premature stop codons, it was regarded as a pseudogene.

#### 2.1.3. Gene Nomenclature

The names of ORs and GRs were designated as per the nomenclature conventions of *B. mori*, *H. melpomene*, *M. sexta* and *H. armigera* [26,31,39,47,48,51,52], coupled with the physical location on scaffolds. Based on the homology, this conserved Orco was named alone. Other ORs were assigned the names of OR1–OR60. For the GR gene family, GR1–GR3 were defined as CO_2_ receptors; GR4–GR8, GR10 and GR11 were sugar receptors; GR9, GR12 and GR13 were GR43a-like receptors; and the remaining GRs (GR14–GR51) were bitter GRs. If one gene within an orthologous group had two or more copies, their names were assigned with copy numbers, for example, OR39.1–OR39.4 in *P. polytes* (Table S1).

#### 2.1.4. Intron Analysis and Genomic Arrangement

In the analyses of the gene structure of ORs and GRs, we summarized the numbers, phases and sizes of introns using GeneWise [50]. Accordingly, intron gains and losses among orthologous genes were analyzed and compared, coupled with the phylogeny. In order to present the distribution of genes on scaffolds and typical gene duplications, we mapped ORs or GRs onto their respective reference genomes. After that, information on scaffold distribution, transcriptional orientation and positions of ORs and GRs was analyzed.

#### 2.1.5. Phylogenetic Analysis

In order to unravel the orthologous relationships of ORs or GRs in six *Papilio* butterflies as well as the correlation of their intron numbers in phylogeny, we constructed four trees. Firstly, only full-length ORs (322 genes) or GRs (271 genes) were selected to build the trees for analyzing the phylogenetic correlation of intron numbers. Secondly, in the classification of OR or GR orthologous groups, all amino acid sequences, excluding pseudogenes, were used, representing 374 ORs and 326 GRs from six *Papilio* species. Multiple alignments of the protein sequences were performed using MAFFT v7.450, applying the FFT–NS–2 algorithm and default parameters (scoring matrix: BLOSUM62, gap open penalty: 1.53 and offset value: 0.123) [53]. The maximum-likelihood trees were inferred by FastTree v2.1.11 under the Whelan and Goldman (WAG) model with SH-like 1000 support [54]. The trees were further visualized and edited using FigTree v1.4.4 (http://tree.bio.ed.ac.uk/software/figtree/, accessed on 12 March 2022).

### 2.2. Molecular Biology

#### 2.2.1. Insect Rearing and Tissue Collection

The pupae of *P. xuthus* were obtained from an insect farm in Suqian City, Jiangsu Province, China. After adult eclosion, they were sexed and separately kept in different cages with 10% sugar water and fermented orange juice. In order to determine the expression profiles of ORs and GRs, various tissues from 3-day-old female and male butterflies were collected, including antennae, proboscises, heads without antennae and proboscises, thoraxes, abdomens without reproductive systems, legs and wings, as well as reproductive-related tissues (i.e., accessory glands, spermatheca connecting spermathecal glands, ovaries and bursa copulatrix of females; accessory glands, ejaculatory ducts, seminal vesicles and testes of males). Each tissue was prepared for at least three biological pools. All these tissues were immediately frozen in liquid nitrogen and stored at −80 °C.

#### 2.2.2. Total RNA Extraction and First-Strand cDNA Synthesis

The tissues were ground using the glass homogenizers by adding 1 mL TRIzol reagent (Invitrogen, Carlsbad, CA, USA), along with the manufacturer’s instructions. A NanoDrop 1000 Spectrophotometer (Thermo Fisher Scientific, San Jose, CA, USA) and agarose gel electrophoresis (1%, *w*/*v*) were used to estimate the concentration and quality of RNA samples. In order to eliminate the effects of genomic DNA (gDNA) on expression profiles of chemosensory receptors, gDNA Eraser in a PrimeScript™ RT reagent Kit (TaKaRa, Dalian, Liaoning, China) was used to digest gDNA at 42 °C for 2 min. Next, 1 μg of RNA was used to synthesize cDNA templates, following the procedure: 37 °C for 15 min and 85 °C for 5 s.

#### 2.2.3. Expression Profiling Analysis of ORs and GRs in *P. xuthus*

In order to investigate the expression of OR and GR genes in different tissues of *P. xuthus*, a reverse transcription PCR (RT–PCR) assay was first employed to examine their tissue distribution. In the analyses, to further improve the accuracy and reliability, amplification fragments comprised at least one intron if possible. Gene-specific primers were designed using Primer Premier 5 (PREMIER Biosoft International, Palo Alto, CA, USA), with product sizes of 400–600 bp. A control gene, ribosomal protein S4 (PxutRPS4), was used to examine the quality and quantity of templates. The reactions were conducted with a Taq DNA Polymerase Kit (TaKaRa, Dalian, Liaoning, China) under thermal cycle conditions: 94 °C for 3 min; 35 cycles at 94 °C for 30 s, 60 °C for 30 s and 72 °C for 40 s; and final extension at 72 °C for 5 min. The amplification products were analyzed by 1.2% (*w*/*v*) agarose gels. Two independent biological templates were run, with one replicate for each template. If the results of two replicates were inconsistent, the third biological template was required. The primer sequences of ORs and GRs are listed in Table S2.

Based on RT–PCR results, we randomly selected 19 OR and 13 GR genes to detect their relative expression levels in tissues, representing antennae, abdomens and reproductive tissues for ORs, as well as proboscises and reproductive tissues for GRs. Quantitative real-time PCR (qPCR) was run on a qTOWER 2.2 instrument (Analytik Jena AG, Jena, Germany), using Bestar^®^ SybrGreen qPCR Mastermix (DBI^®^ Bioscience, Germany) and gene-specific primers designed by Beacon Designer 8.14 (PREMIER Biosoft International, Palo Alto, CA, USA). Relative expression of receptor genes was computed relative to a control gene, PxutRPL8 [42], using a Q-Gene package [55,56]. Three biological replicates were conducted for each gene, three with each template. The primer sequences of candidate genes are listed in Table S2. Statistical significant differences among tissues were analyzed using one-way ANOVA followed by Fisher’s LSD test (*p* < 0.05), implemented in IBM SPSS Statistics 21 (SPSS Inc., Chicago, IL, USA).

## 3. Results

### 3.1. Identification of Papilio ORs and GRs

**Figure 1:**
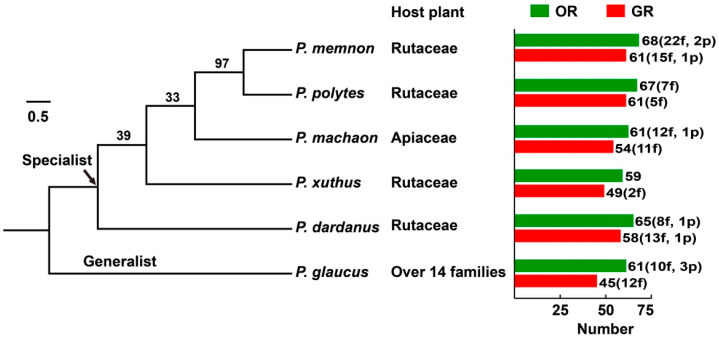
The OR and GR gene families in six *Papilio* butterflies. The tree was constructed with MEGA 11, based on an aligned Orco protein sequence. On the right of this figure, the numbers of genes encoding ORs (green) and GRs (red) are indicated. Numbers in parentheses represent fragments (f) and pseudogenes (p).

By using a homology-based BLAST search, a total of 381 genes encoding ORs were identified, including six Orco receptors, 22 PRs and seven pseudogenes, ranging from 59 relatives in *P. xuthus* to 68 in *P. memnon*. Of these, 322 sequences were predicted to have complete open reading frames (ORFs), of which this coreceptor Orco harbored the longest sequence in each species (*P. memnon* Orco: 1443 bp and other five Orco genes: 1425 bp). The remaining 59 genes were partial sequences, representing the largest number of 22 in *P. memnon*. Out of the 59 OR fragments, the sequences varied from 162 bp in PmemOR42 to 1344 bp in PmacOR40. Notably, some fragments were subjected to premature stop codons and defined as pseudogenes, including PdarOR39.3/PglaOR41.2/PglaOR53.2/PglaOR59/PmacOR25.1/PmemOR24.1/PmemOR60.2 (Figure 1 and Table S1).

The numbers of GRs identified from *P. glaucus*, *P. xuthus*, *P. machaon*, *P. dardanus*, *P. polytes* and *P. memnon* were 45, 49, 54, 58, 61 and 61, respectively. Of the identified GRs, 271 genes had complete full-length sequences with the longest ORFs for GR3 and GR5 members. Other 57 GRs were identified as fragments, encoding 157 (PdarGR39) to 480 (PmacGR5) amino acids. Two candidate GRs, PdarGR4.2 and PmemGR44.2, were identified as pseudogenes. As *P. xuthus* GR1 was described in a previous study [29], it was renamed PxutGR13 based on the GR nomenclature conventions in Lepidoptera. The orthologs of PxutGR13 were also identified in other five *Papilio* species, with two gene copies in *P. memnon* (PmemGR13.1 and PmemGR13.2) and *P. polytes* (PpolGR13.1 and PpolGR13.2) (Figure 1 and Table S1).

### 3.2. Intron Analysis of Papilio ORs and GRs

**Figure 2:**
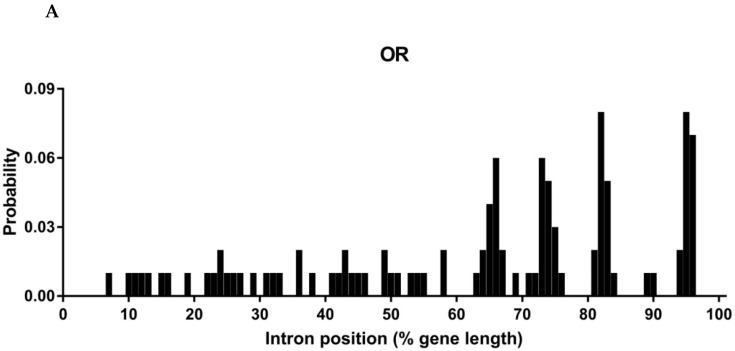

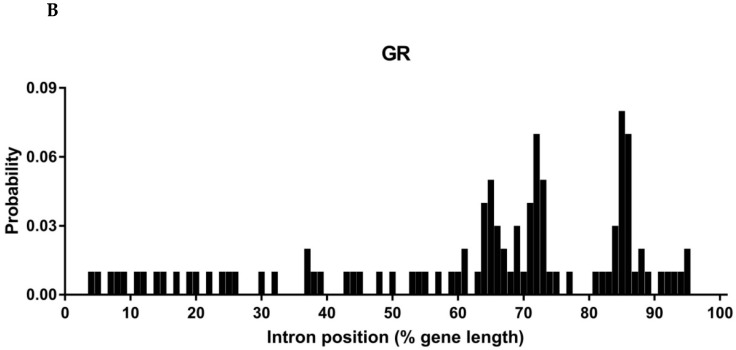
The distribution of intron positions with a percentage of gene length for full-length ORs (**A**) and GRs (**B**) in six *Papilio* butterflies.

Differences in gene structure provide important information on the evolution of gene families, including intron numbers, insertion sites, phases and sizes. In general, orthologous chemoreceptor genes across insects have conserved gene structures [57,58,59]. As expected, most orthologous ORs or GRs exhibited the same intron numbers, insertion sites and phases, as well as similar intron sizes. Taking Orco genes as an example, they had nine introns and identical intron phases (1–2–1–0–1–2–0–0–0) in all six species. Similarly, GR2 genes comprised eight introns and the same phases of 0–1–0–0–2–0–0–0 (Table S1). By analyzing insertion positions of introns, the majority of introns were located on the latter parts of genes, i.e., 60%–100% of regions of an OR or GR gene (Figure 2A,B).

**Figure 3:**
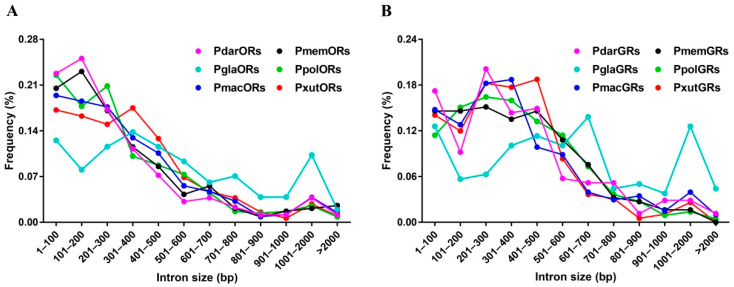
The frequency of intron sizes for full-length ORs (**A**) and GRs (**B**) in six *Papilio* butterflies.

In order to reveal the possible relationships between intron sizes and the host range in six *Papilio* butterflies, ORs or GRs with full-length ORFs were analyzed. In the OR gene family, five specialists had similar frequencies of intron sizes, of which the majority of intron lengths ranged from 1 to 600 bp. Remarkably, it was noticed that this generalist *P. glaucus* showed a different frequency pattern of intron lengths relative to the specialists (Figure 3A). A similar phenomenon on the frequency of intron sizes was also presented in the GR gene repertoire, as *P. glaucus* had more variable intron lengths than five other *Papilio* specialists (Figure 3B).

### 3.3. Phylogenetic Analysis of Papilio ORs

**Figure 4:**
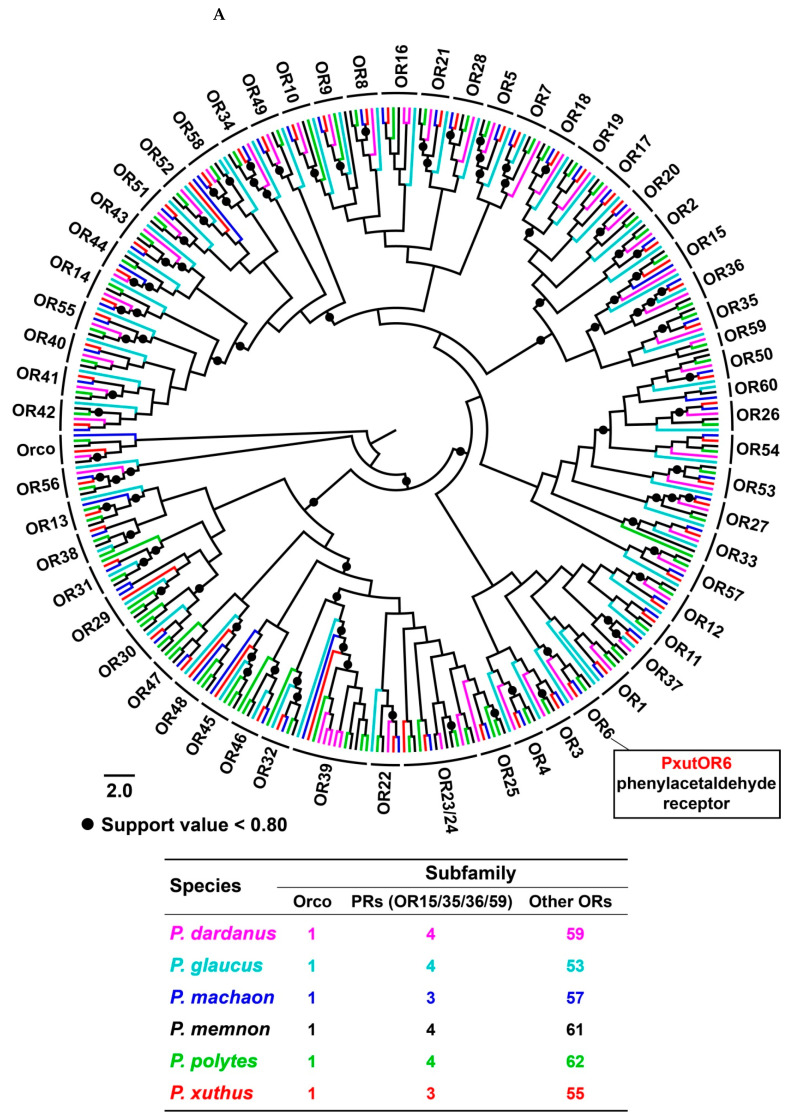

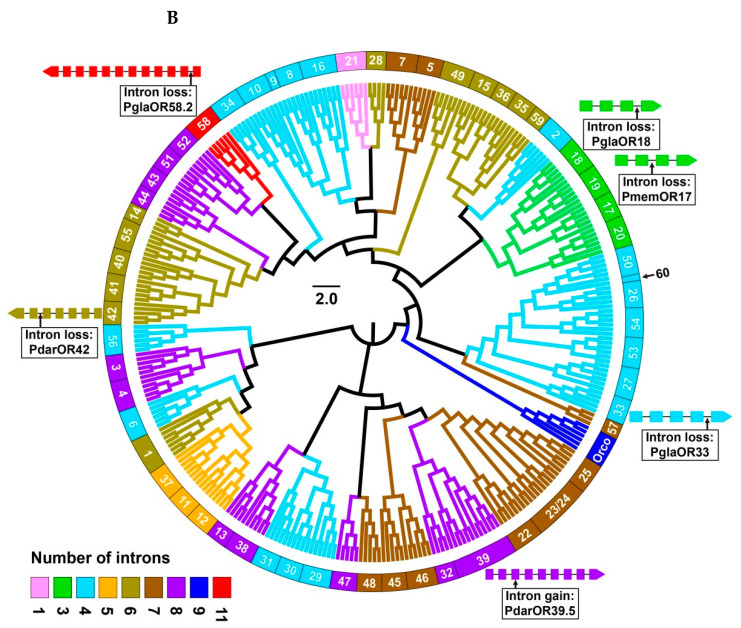
The OR gene family in six *Papilio* butterflies. (**A**) Maximum likelihood tree of ORs in six *Papilio* species, based on aligned protein sequences of 374 ORs. A total of 60 orthologous groups (Orco, OR1–OR23/24 and OR25–OR60) were classified based on the orthology. Four candidate PR groups (OR15/OR35/OR36/OR59) clustered into a large clade. (**B**) A correlation analysis of ORs showing the relationships between intron numbers and orthologous groups in six *Papilio* species. This analysis was performed with 322 full-length ORs. Intron gains and losses of ORs are indicated by the side of orthologous groups.

With an aligned protein sequence of 374 ORs from six *Papilio* species, we constructed a maximum likelihood tree using FastTree *v*2.1.11. Following the classification of ORs in Lepidoptera, *Papilio* ORs were composed of three subfamilies, i.e., Orco, PRs and other ORs that were further divided into 60 orthologous groups (Orco, OR1–OR23/24 and OR25–OR60). This Orco clade shared a high degree of conservation (a mean amino acid identity of 97.61%) across six *Papilio* species, with a high support value (100%) and 1:1:1:1:1:1 orthology. Such one-to-one orthologous relationships among six species were presented in other 44 OR groups, including three of four PR clades (OR15/OR35/OR36) as well as putative phenylacetaldehyde-sensitive OR6s (Figure 4A). The remaining 15 orthologous groups had more or fewer gene gains or losses of ORs. For example, *P. dardanus* possessed six gene copies of OR39. Four paralogs in OR23 and OR39 groups were identified from *P. memnon* and *P. polytes*, respectively. In addition, some species lost several OR orthologs, especially *P. glaucus,* where four clades (OR23–OR25 and OR47) did not comprise PglaORs. Members of a candidate PR clade OR59 were absent in *P. machaon* and *P. xuthus* (Table 1).

Focusing on the correlation between intron numbers and orthologous groups, we categorized introns of *Papilio* ORs into nine types according to the numbers (1, 3, 4, 5, 6, 7, 8, 9 and 11). In the tree, it was noticed that each orthologous group harbored the same number of introns, with the exception of six clades (OR17/OR18/OR33/OR39/OR42/OR58). PdarOR39.5 in *P. dardanus* gained an additional intron between the second and third introns (up to nine). The other five ORs lost one intron, including PglaOR18, PglaOR33, PglaOR58.2, PmemOR17 and PdarOR42. The genes containing the same intron numbers did not cluster into a large clade. In contrast, they were distributed into different clades (Figure 4B).

### 3.4. Phylogenetic Analysis of Papilio GRs

**Figure 5:**
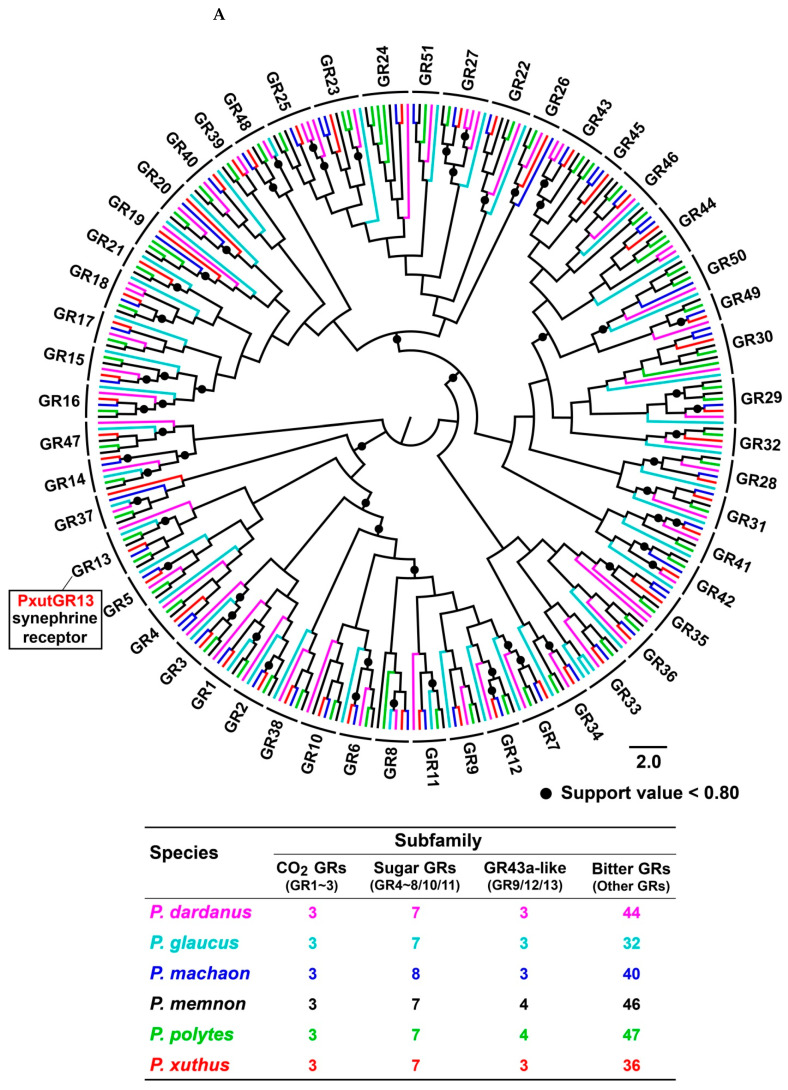

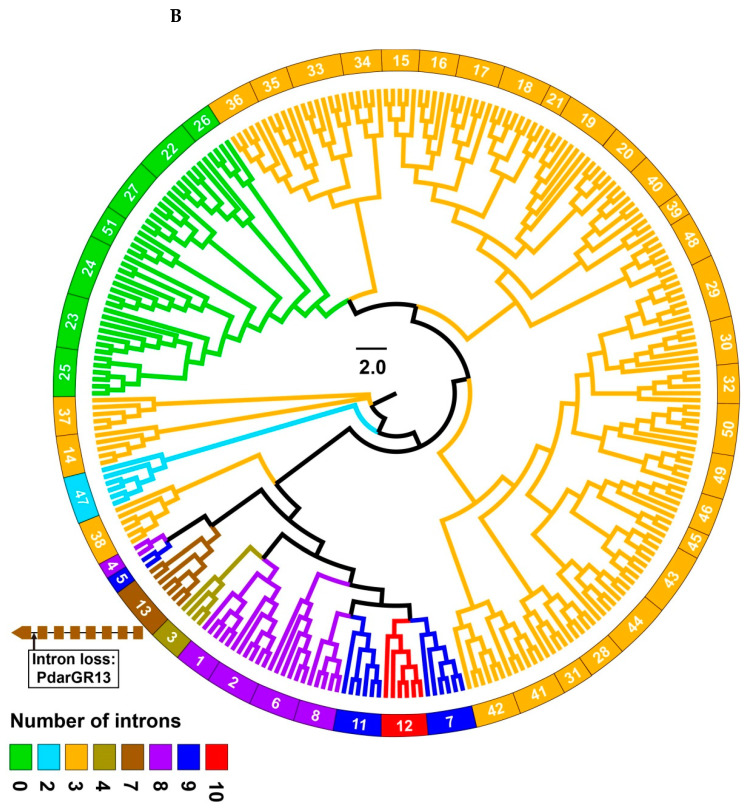
The GR gene family in six *Papilio* butterflies. (**A**) Maximum likelihood tree of GRs in six *Papilio* species, based on aligned protein sequences of 326 GRs. A total of 51 orthologous groups (three CO_2_ GRs, seven sugar GRs, three GR43a-like GRs and 38 bitter GRs) were classified based on orthology. (**B**) A correlation analysis of GRs showing the relationships between intron numbers and orthologous groups in six *Papilio* species. This analysis was performed with 271 full-length GRs. An intron loss of PdarGR13 in *P. dardanus* is indicated by the side of the orthologous group.

In the phylogenetic analysis, 326 *Papilio* GRs were classified into 51 orthologous groups (GR1–GR51). Three subfamilies of GRs, including CO_2_, sugar and GR43a-like receptors, were highly conserved, with 11 clades showing one-to-one relationships among six species. In the bitter receptor subfamily, 15 orthologous groups comprised a strict single gene from each species, including a synephrine receptor PxutGR13 and its orthologs (Figure 5A). Apart from the above 26 groups, the remaining 24 clades had variable gene numbers among six species. Half of the 24 clades contained 1–3 genes in six species, as a representative of gene gains. The other 12 groups presented gene gains or losses of GRs in some species. For example, *P. glaucus* lacked eight GRs in 12 orthologous groups. In comparison, *P. dardanus* and *P. polytes* gained 10 and 9 genes in 24 groups, respectively (Table 1).

*Papilio* GRs had eight types of introns, ranging from 0 to 10 categories. Differing from the OR gene family in *Papilio*, the majority of GRs with the same intron numbers clustered together. In the bitter receptor subfamily, they had low intron numbers (0–3). For the intronless GRs, a relatively large clade was formed by seven groups (GR22–GR27 and GR51). Twenty-seven GR groups with three introns clustered into a large clade close to the intronless GRs. On the other hand, three conserved GR subfamilies harbored more intron numbers compared to bitter GRs and were phylogenetically grouped into a clade (Figure 5B).

### 3.5. Genomic Arrangement of Papilio ORs and GRs

**Figure 6:**
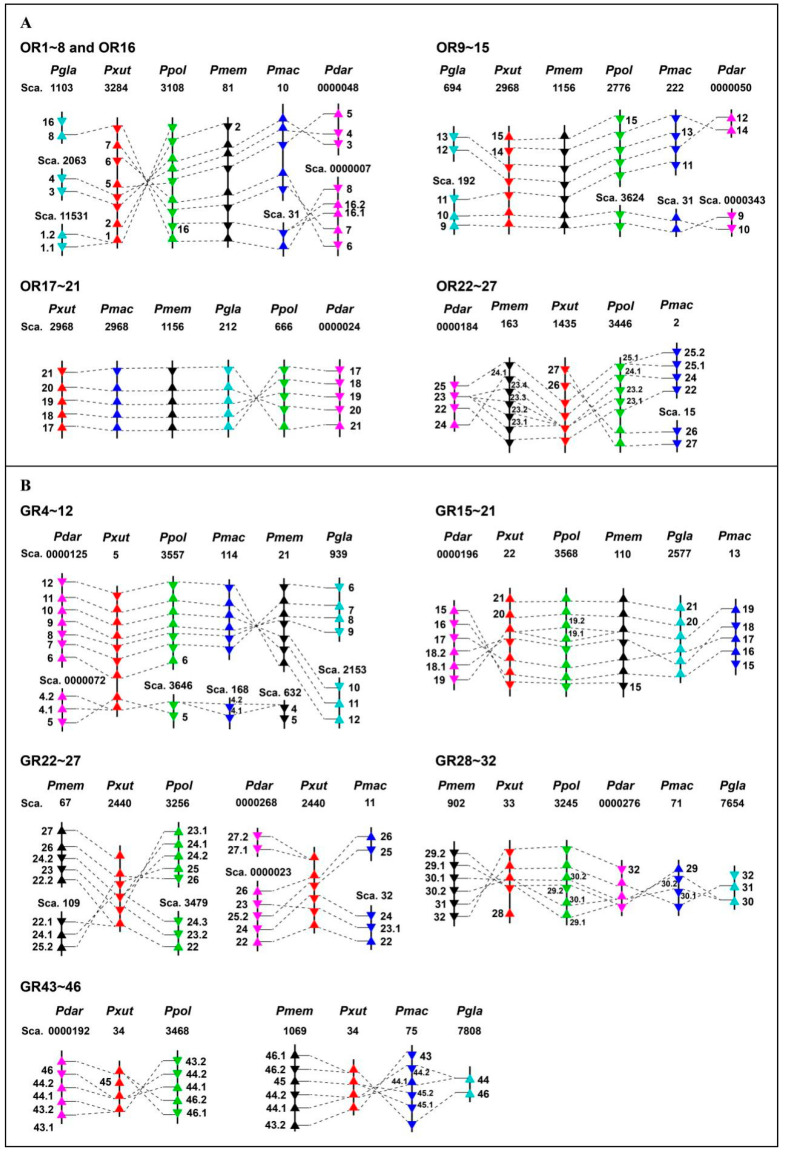
Genomic arrangement and the orthology of ORs (**A**) and GRs (**B**) in six *Papilio* butterflies. When two or more genes in one species are situated on one scaffold, they are indicated with the transcriptional orientation. Based on the phylogeny, orthologous genes within each cluster were linked by dotted lines. Pdar, *P. dardanus*; Pgla, *P. glaucus*; Pmac, *P. machaon*; Pmem, *P. memnon*; Ppol, *P. polytes* and Pxut, *P. xuthus*.

Based on the phylogeny, we analyzed typical gene clusters on scaffolds and their orthology among six *Papilio* butterflies. For the OR gene repertoire, four clusters were found, representing OR1–OR8/OR16, OR9–OR15, OR17–OR21 and OR22–OR27. Of these, this OR17–OR21 cluster had the highest conservation among six species, of which five genes were tandemly situated on one scaffold in each butterfly. OR genes in the other three clusters were located on one or more scaffolds in close proximity and had relatively high conservation among these *Papilio* species (Figure 6A).

In the clustering distribution of *Papilio* GRs, five groups were identified, including a sugar receptor subfamily (GR4–GR12). Nine sugar GRs from *P. xuthus* were arrayed on scaffold 5, but their orthologous genes in the other five species were distributed on at least two scaffolds. The genes of four bitter GRs clusters were mapped to at least one scaffold, as some orthologs were absent or had more gene copies (Figure 6B).

### 3.6. Expression Profiling Analysis of P. xuthus ORs

**Figure 7:**
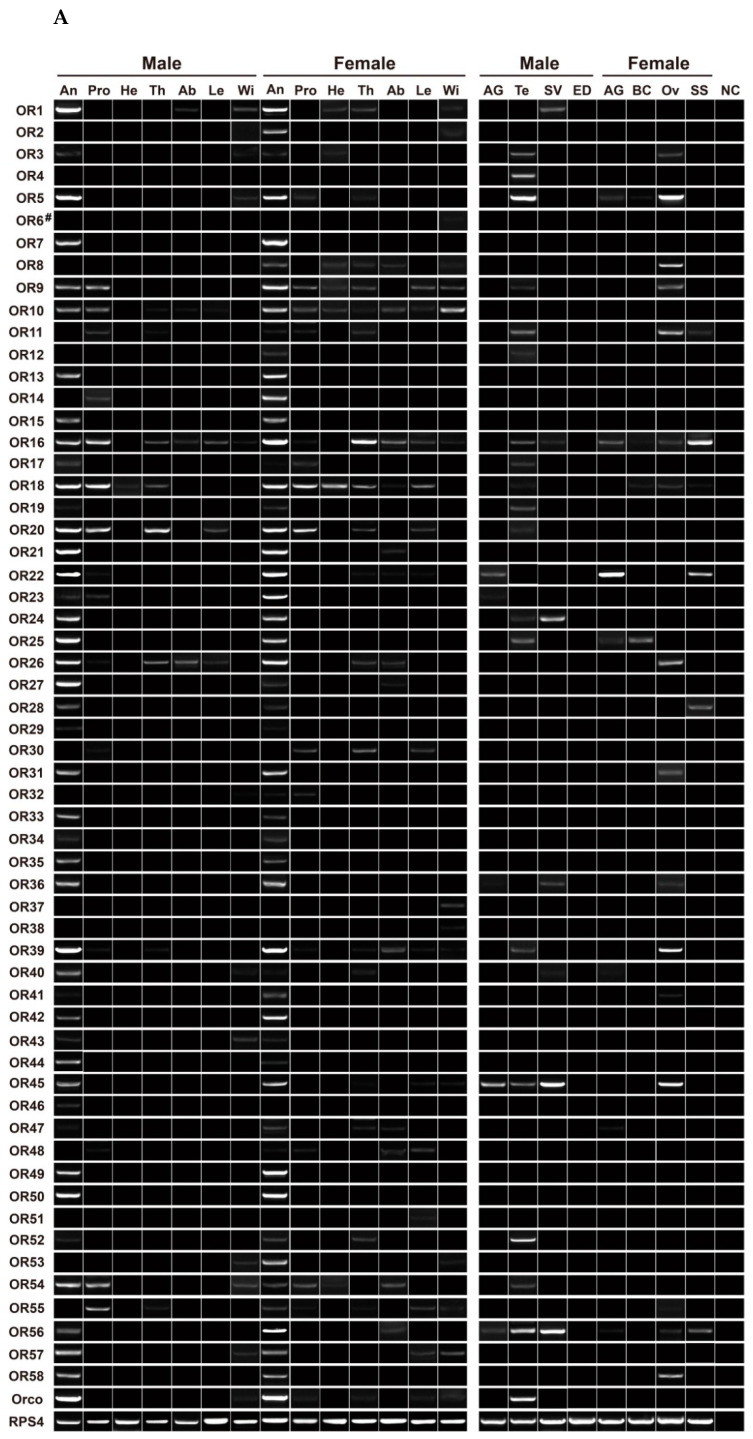

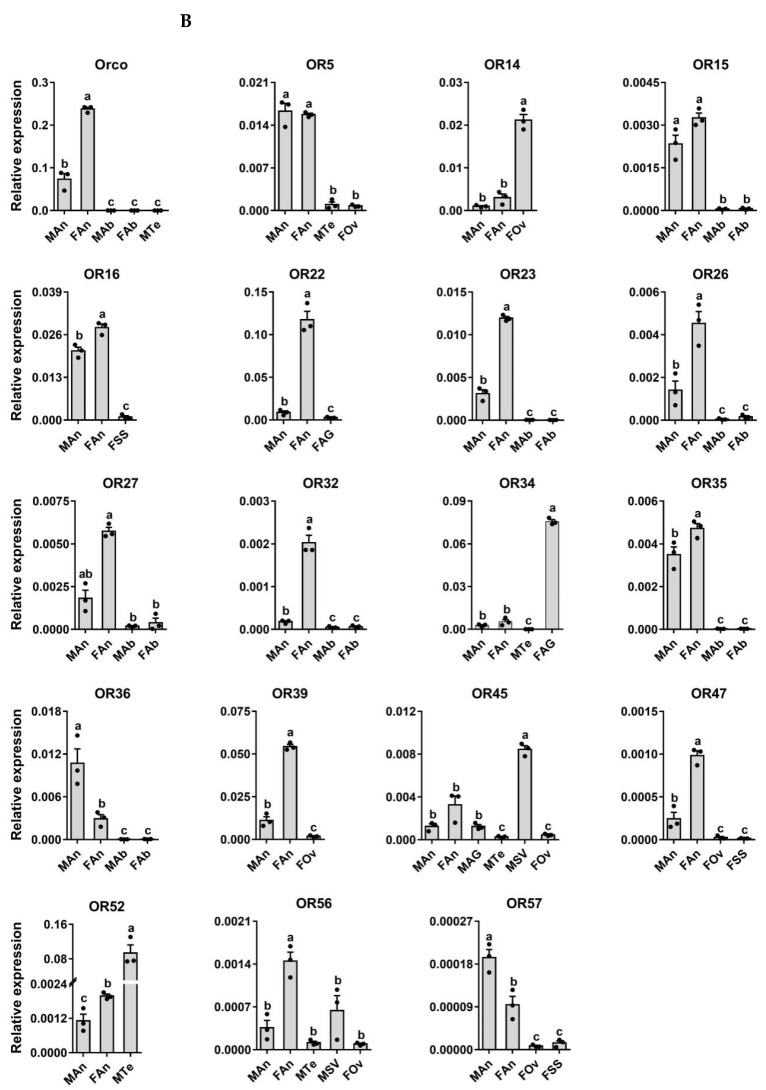
Expression profile of OR genes in various tissues of *P. xuthus*. (**A**) Expression of 59 ORs in different tissues. “#” denotes this gene has no detectable expression in all tested tissues. (**B**) Relative expression of 19 ORs in antennae, abdomens and reproductive tissues. The expression data of ORs in each tissue are indicated by scatter plots, with three biological replicates. Different lowercases on error bars represent statistical significance (ANOVA, LSD, *p* < 0.05). An, antennae; Pro, proboscises; He, heads without antennae and proboscises; Th, thoraxes; Ab, abdomens; Le, legs; Wi, wings; AG, accessory glands; SS, spermatheca connecting spermathecal glands; Ov, ovaries; BC, bursa copulatrix; ED, ejaculatory ducts; SV, seminal vesicles; Te, testes and NC, negative control using sterile water as the templates.

Out of the six *Papilio* butterflies, we chose *P. xuthus* to study the expression profiles of 59 PxutORs in 14 adult tissues and eight reproductive tissues of both sexes. Except for PxutOR6, the expression of all 58 PxutOR genes was detectable in at least one tissue. The majority of genes had specific or high expression in female and male antennae, accounting for 89.66% (52/58) of the genes. By focusing on antenna-exclusive genes, nine PxutORs were found to have such an expression, including PxutOR2/OR7/OR13/OR33–OR35/OR44/OR49/OR50. Moreover, some genes appeared to exhibit sex-biased levels in the antennae, such as PxutOR2/OR8/OR23/OR42/OR53 in females and PxutOR27/OR40/OR44 in males. Outside the antennal tissue, a certain number of genes were expressed in proboscises, legs and non-chemosensory tissues. In particular, 31 genes had detectable expression in at least one reproductive tissue. This coreceptor PxutOrco and three candidate PxutPRs (PxutOR15/OR35/OR36) were enriched in the antennae (Figure 7A).

By using the qPCR assay, we further measured the relative expression of 19 PxutORs in antennae and non-chemosensory tissues. Most PxutORs (15/19) were significantly enriched in female and/or male antennae. PxutOrco displayed a significant female-biased expression in antennae compared to males, with a 4.90-fold difference. Three candidate PR genes showed antenna-predominant expression, of which PxutOR35 was a female-biased gene while PxutOR36 exhibited a significant level in males. Out of the remaining 15 genes, five (PxutOR5/OR14/OR27/OR34/OR45) had almost equal expression in the antennae of both sexes, nine (PxutOR16/OR22/OR23/OR26/OR32/ OR39/OR47/OR52/OR56) for female-antenna-biased expression and PxutOR57 for a male-antenna-biased level. Notably, four genes were found to have particularly high expression in reproductive-related tissues, such as PxutOR14 in female ovaries (6.34-fold higher than female antennae), PxutOR34 in female accessory glands (9.68-fold higher than female antennae), PxutOR45 in male seminal vesicles (1.89-fold higher than female antennae) and PxutOR52 in male testes (38.00-fold higher than female antennae) (Figure 7B).

### 3.7. Expression Profiling Analysis of P. xuthus GRs

**Figure 8:**
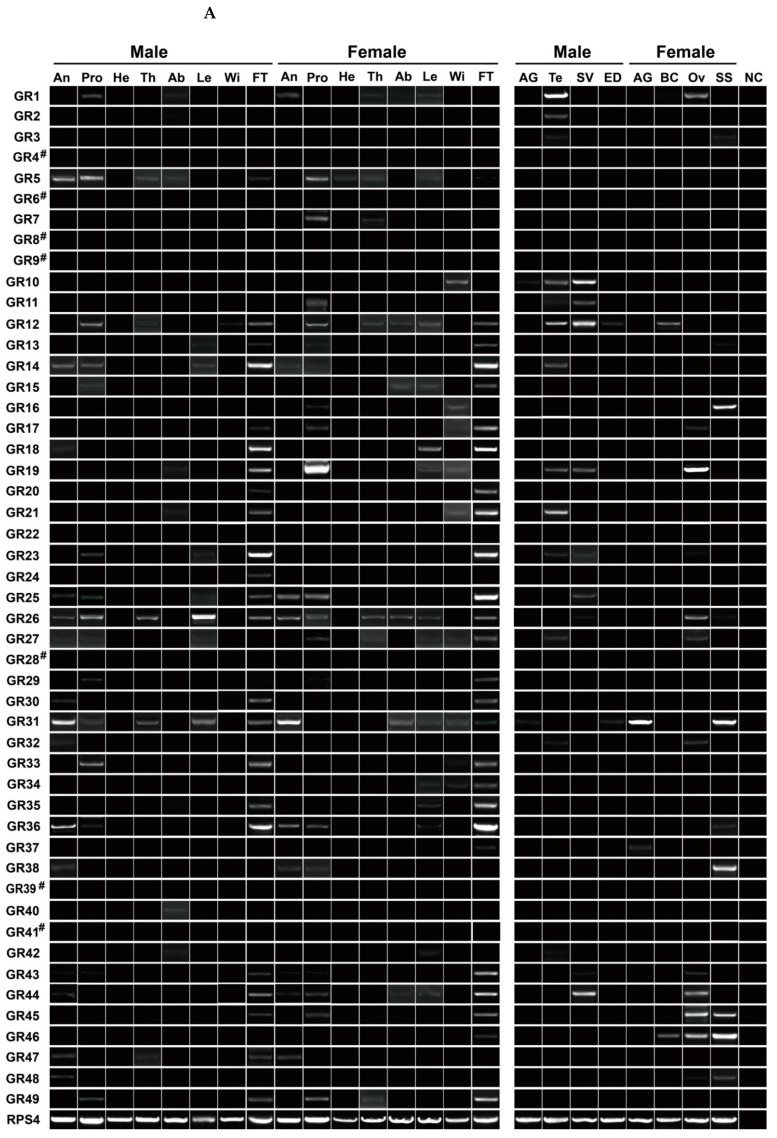

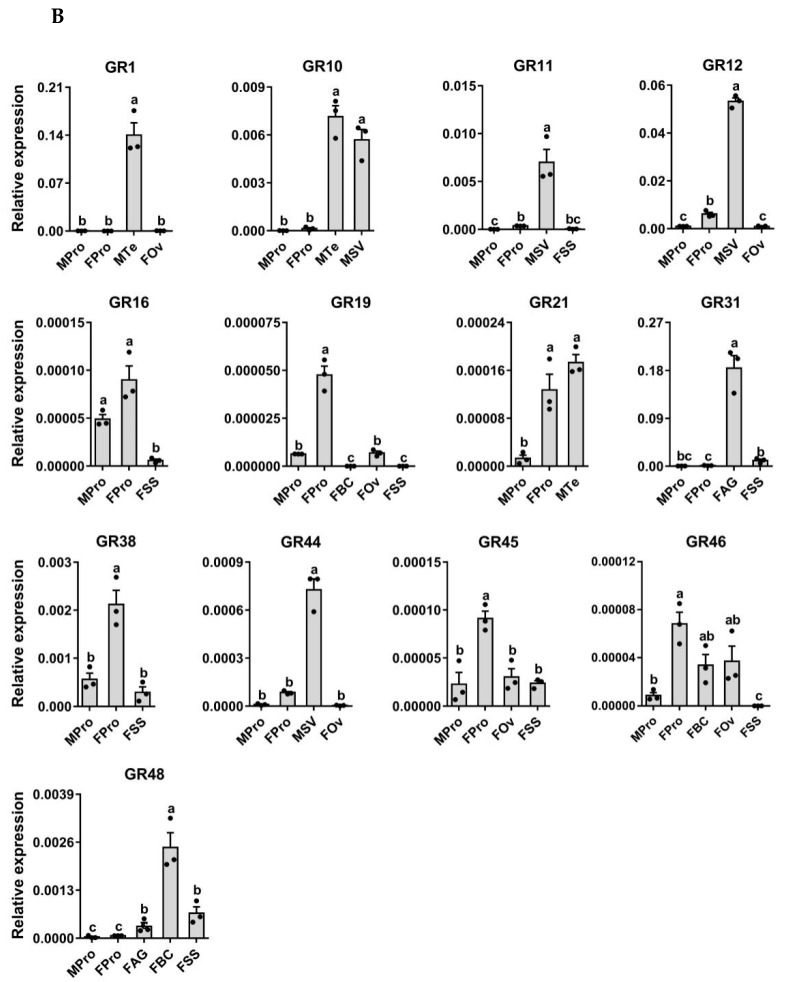
Expression profile of GR genes in various tissues of *P. xuthus*. (**A**) Expression of 49 GRs in different tissues. “#” denotes the genes have no detectable expression in all tested tissues. (**B**) Relative expression of 13 GRs in proboscises and reproductive tissues. The expression data of GRs in each tissue are indicated by scatter plots, with three biological replicates. Different lowercases on error bars represent statistical significance (ANOVA, LSD, *p* < 0.05). FT, foretarsi and NC, negative control using sterile water as the templates. The abbreviation of other tissues is shown in Figure 7.

The female *P. xuthus* use their foreleg tarsi to drum the surface of the leaves of host plants before laying eggs. As a contact chemoreception behavior, taste-associated genes such as GRs are key molecular candidates in foreleg tarsi [29,60]. Hence, we additionally collected foreleg tarsi of both sexes to investigate the expression of 49 PxutGRs in 24 tissues. Seven of them (PxutGR4/GR6/GR8/GR9/GR28/GR39/GR41) had no detectable expression in all tested tissues. Of the remaining 42 genes, as many as 28 relatives were expressed in foreleg tarsi, where 11 genes appeared to be female-biased relatives. Additionally, 9 and 19 genes were detected separately in antennae and proboscises, although most of them showed extremely low expression levels. Similar to ORs, a comparable number of GRs had the expression in reproductive tissues, summing to 25 genes expressed in at least one tissue (Figure 8A).

In qPCR analyses of 13 PxutGRs, all the genes were detected in reproductive tissues, consistent with RT–PCR results. Four conserved GRs were enriched in reproductive tissues, such as a CO_2_ receptor PxutGR1 in male testes, two sugar receptors PxutGR10 and PxutGR11 in male testes and/or male seminal vesicles and a GR43a-like receptor PxutGR12 in male seminal vesicles. Out of nine bitter receptor genes, PxutGR16/GR19/GR38/GR45 were expressed in proboscises at a significant level. Among tested tissues, PxutGR31, GR44 and GR48 had the highest expression in female accessory glands, male seminal vesicles and female bursa copulatrix, respectively (Figure 8B).

## 4. Discussion

The *Papilio* butterfly is one of the most suitable herbivores for investigating insect–plant chemical interactions. In the present study, we addressed the issue of whether the host plant range shapes the evolution of chemoreceptors in *Papilio* species associated with smell (ORs) and taste (GRs) outside previously characterized ionotropic receptors [42,57], coupled with selective pressure from the surrounding environment. In the genus *Papilio*, *P. glaucus* is known as a representative of the most polyphagous species and thus is included in our analyses [5]. The other five *Papilio* butterflies were randomly selected and defined as specialists because their larvae feed solely on the plants in a family or a genus [4] (see Figure 1). Unlike moths as agricultural and forest pests, the majority of these butterflies received relatively little attention on the genetic and molecular basis of chemoreception. Hence, our study represents, for the first time, a comprehensively comparative analysis of ORs and GRs in Papilionidae, facilitating the understanding of herbivorous insects and host plants coevolution.

The expansion of chemosensory-related gene families was suggested to be correlated with diverse host plants, particularly the GR gene repertoire. In polyphagous noctuid moths *H. armigera*, *S. litura*, *S. frugiperda*, *Spodoptera littoralis* and *Agrotis ipsilon*, they possessed a large number of GRs in response to plant secondary metabolites (i.e., bitter tastants), namely bitter receptors ranging from 165 relatives in *A. ipsilon* to 294 in *S. littoralis* [23,24,39,40,61]. Differing from the mechanism of GR expansions in polyphagous moths by gene duplications, the generalist *Anoplophora glabripennis* in Coleoptera gained more GR genes via a combination of tandem duplications and alternative splicing compared to two specialists, *Agrilus planipennis* and *Dendroctonus ponderosae* [20,62]. Such a large-scale expansion possibly reflects an evolutionary adaptation of herbivorous insects to numerous feeding or oviposition hosts. In six *Papilio* butterflies, some bitter GR clusters were observed, possibly derived from gene duplication. Exceptionally, the generalist *P. glaucus* had a slightly contractive GR repertoire relative to five other *Papilio* specialists. In the analyses of GR gains and losses, as well as the phylogeny of GRs, we noticed that the eastern tiger swallowtail butterfly lost eight GR orthologs and retained single-copy genes in the remaining groups but not including GR33. This contraction of the GR repertoire in this polyphagous butterfly was uncommon, as other polyphagous herbivores had similar or more GRs than those species feeding exclusively on a limited number of plants [31]. For instance, the generalist *V. cardui* harbored 50 GRs, whereas the GR numbers of three specialists belonging to the same family varied from 17 to 45 genes [10]. In our study, the smallest number of *P. glaucus* GRs was possibly attributed to the quality of genome sequencing and assembly. The *P. glaucus* had the lowest scaffold N50 value (0.23 Mb) among six *Papilio* species (*P. dardanus*: N50 = 0.60 Mb; *P. machaon*: N50 = 1.15 Mb; *P. polytes*: N50 = 3.70 Mb; *P. memnon*: N50 = 5.50 Mb and *P. xuthus*: N50 = 6.20 Mb) [41,43,44,45,46]. Additionally, it was reasonably inferred that the number of such host plants in *P. glaucus* (14 families) is insufficient to result in the expansion of GRs in comparison to other polyphagous moths or beetles with over 100 hosts [5,25,63,64]. For the five *Papilio* specialists, they use a limited number of plants as food sources [3,4]. The sizes of their GR repertoires (49–61 relatives) were similar to those in the monophagous or oligophagous herbivores *B. mori* (76 GRs) [47], *M. sexta* (45 GRs) [26], *P. xylostella* (69 GRs) [11], *D. plexippus* (59 GRs) [49] and *H. melpomene* (73 GRs) [48].

In comparison, the sizes of OR repertoires among the Lepidoptera (69 ORs in *S. frugiperda* to 86 in *A. ipsilon*) or the Coleoptera (47 ORs in *A. planipennis* to 132 in *A. glabripennis*) were relatively stable [20,23,40,62]. In six *Papilio* butterflies, the generalist and five specialists harbored similar OR numbers (59–68 relatives), further supporting the notion that host range differences do not lead to the large-scale expansions of the OR repertoires in herbivorous insects as observed in two butterflies *D. plexippus* and *H. melpomene* [48,49]. Similar to the monarch-specific expansions of ORs in *D. plexippus* [49], some *Papilio* OR genes clustering into one scaffold in a butterfly may be produced by gene duplications. Ecologically, the olfactory system of butterflies or moths not only detects host plant volatiles but is also associated with the perception of other volatile compounds produced by non-host plants, animals, microbes and human activities. Therefore, it is reasonable that the evolution of ORs in Lepidoptera may be little shaped by the host plant range.

In Lepidoptera, the evolution of ORs or GRs may shape their gene structure as the orthologous group or the same subfamily shares conserved numbers, insertion sites and phases of introns, especially for the GR gene family [39,47,58]. In all six *Papilio* butterflies, it appeared that host plant differences did not apparently shape the evolution of intron numbers, insertion sites or phases, consistent with the results in other lepidopteran species [39,58]. Nevertheless, it was noticed that ORs of the generalist *P. glaucus* had a more variable frequency pattern of intron lengths relative to those in five *Papilio* specialists, as did the GR gene family. This variation in ORs or GRs in Papilionidae may be correlated with the host range, as the main difference between *P. glaucus* and the other five *Papilio* specialists occurred in the breath of host plants. With a number of available genomes in Lepidoptera, an extensive analysis of gene structure will provide insights into the relationships between intron sizes and the diversity of host plants. Similar to other lepidopteran species, *Papilio* GRs were mainly constituted of GRs with a few introns (0–3), representing all bitter receptor members (38 orthologous clades). As indicated in the function that bitter GRs in Lepidoptera mainly responded to plant secondary compounds [30,37,38,39,65], a relatively simple gene structure may be more favorable for the expansion of this subfamily and the rapid coevolution of herbivorous insects to the constantly expanding host plants. This hypothesis was further supported as three conserved GR subfamilies (i.e., CO_2_, sugar and GR43a-like GRs) had complex gene structures and few gene gains among lepidopteran species [31,51,52], including *Papilio* butterflies in this study.

As indicated in other lepidopteran species, a number of bitter GRs shared three introns in conserved positions located at the latter regions of sequences and the same phase of 0–0–1 [11,58]. Such the feature of introns also occurred in the GR gene family of six *Papilio* butterflies but was different from non-lepidopteran species *Apis mellifera* [66] and *Drosophila melanogaster* [67]. The intron patterns of *Papilio* GRs further supported the hypothesis that lepidopteran GRs were derived from only a few common ancestors [11]. On the other hand, we detected several intronless GR groups in *Papilio* species, consistent with most *H. armigera* GRs but different from the majority of *S. litura* GRs [24,25]. These findings suggested different mechanisms underlying the expansions of GRs across the Lepidoptera. In comparison to GRs, *Papilio* ORs had more variable intron numbers, similar to those in other Lepidoptera [11] and Hemiptera [21]. Intriguingly, a comparable number of *Papilio* ORs shared four conserved insertion sites of introns with the same phases of 2–0–0–0 near the 3′-terminus. It appeared that this conservation on intron sites and phases of ORs was common across insects, as seen in ORs from *B. mori*, *D. plexippus*, *D. melanogaster* and *Tribolium castaneum* [11,67].

Following the classification of ORs responding to sex pheromones (i.e., PRs) in moths [19,68], our current study identified 3–4 candidate PRs from each *Papilio* species. In the expression profiles of *P. xuthus* PRs, three candidate genes (PxutOR15/OR35/OR36) expectedly showed antenna-specific or high expression but exhibited different sex-biased levels, suggesting different mechanisms underlying the sex pheromone communication with the majority of moths [69]. Out of the six *Papilio* species, potential sex pheromones of only *P. polytes* were identified, representing C10–C16 fatty acids, C23–C27 linear alkenes and C23–C29 linear alkanes [70]. It is of particular significance to study the interactions of four PRs and these components in this species, possibly opening the door for the genetic basis of sex pheromone communication in butterflies. Previously, ORs from 11 species orthologous to HarmOR42 in *H. armigera* had strong activities to a floral scent phenylacetaldehyde, including an ortholog PxutOR6 in *P. xuthus* [71]. Here, we found its orthologs in the other five *Papilio* species, namely the OR6 clade. Between HarmOR42 and six *Papilio* OR6s, 61.99–66.67% amino acid identities were observed. Therefore, it is possible that OR6s in the other five *Papilio* species are also candidate molecular targets for the detection of phenylacetaldehyde, together with particularly high conservation among six *Papilio* OR6s (87.05–94.20% identities).

To seek suitable host plants for oviposition, female butterflies in the genus *Papilio* use their foretarsi to drum the surface of leaves [60,72]. The genes expressed in gustatory sensory neurons of foretarsi such as GRs are key candidates for sensing a variety of oviposition stimulants or repellents. In two butterflies, *P. xuthus* and *P. rapae*, foretarsus-enriched GRs in females could detect oviposition stimulants derived from host plants [29,37]. By focusing on the GRs in *P. xuthus*, a comparable number of genes (25 relatives) were found to have the expression in female foreleg tarsi, suggesting their putative roles in the perception of 10 oviposition stimulants (particularly female-foretarsus-biased GRs) [73]. Notably, a certain number of ORs (including Orco) or GRs in *P. xuthus* had detectable expression in reproductive and non-chemosensory tissues, possibly associated with non-chemosensory functions. In *H. armigera* [25,39,51], *S. litura* [74] and *Achelura yunnanensis* [75], some OR and GR genes were expressed in male testes, female ovaries or other reproductive tissues at a high level, associated with reproduction. Such the putative role of ORs in reproduction was also observed in other insects [76,77,78].

## 5. Conclusions

Our study identified 709 odorant and gustatory receptors in six *Papilio* butterflies, representing the most comprehensive survey in Papilionidae. A correlation analysis between the host range and chemoreceptor gene family sizes revealed the generalist and specialists in *Papilio* species harbored similar gene numbers of ORs or GRs, of which some butterflies presented slight gene gains and losses. Gene structural analyses indicated that orthologous ORs or GRs generally shared conserved intron numbers, phases and insertion sites. The generalist and five specialists had different frequency patterns of intron lengths. Our phylogenetic analyses classified *Papilio* ORs and GRs into 60 and 51 orthologous clades, respectively, of which the GRs with 0–3 introns further clustered into a large clade. Ultimately, we detected the expression of 58 ORs and 44 GRs in tissues, with the majority of ORs in antennae and over half of GRs in female foreleg tarsi. Together, this study comprehensively analyzed and compared the OR and GR gene families in six *Papilio* butterflies and provides reference data for further exploring the roles of ORs and GRs in *P. xuthus* associated with olfaction, oviposition and reproduction.

## Figures and Tables

**Table 1:** Insights into gene gains and losses of *Papilio* ORs and GRs.

Ortholog	Species						Ortholog	Species					
	*Pdar*	*Pgla*	*Pmac*	*Pmem*	*Ppol*	*Pxut*		*Pdar*	*Pgla*	*Pmac*	*Pmem*	*Ppol*	*Pxut*
	*r*	*a*	*c*	*m*	*l*	*t*		*r*	*a*	*c*	*m*	*l*	*t*
OR1/OR41/ OR53/OR58	**1**	**2**	**1**	**1**	**1**	**1**	GR22	**1**	**1**	**1**	**2**	**1**	**1**
OR11		**1**	**1**	**1**	**1**	**1**	GR23	**1**	**1**	**2**	**1**	**2**	**1**
OR16	**2**	**1**	**1**	**1**	**1**	**1**	GR24	**1**		**1**	**2**	**3**	**1**
OR23	**1**			**4**	**2**	**1**	GR25	**3**		**1**	**2**	**1**	**1**
OR24	**1**		**1**	**2**	**2**	**1**	GR26	**1**		**1**	**1**	**1**	**1**
OR25	**1**		**2**	**1**	**2**	**1**	GR27	**3**	**1**	**1**	**1**	**1**	**1**
OR29	**1**	**1**	**2**	**1**	**1**	**1**	GR30	**1**	**1**	**2**	**2**	**2**	**1**
OR30	**1**	**1**	**1**	**2**	**2**	**1**	GR32	**1**	**1**		**1**	**1**	**1**
OR39	**6**	**1**	**1**	**2**	**4**	**1**	GR33	**1**	**3**	**1**	**1**	**1**	**1**
OR47	**1**		**1**	**1**	**1**	**1**	GR35	**2**		**2**	**1**	**1**	**1**
OR59	**1**	**1**		**1**	**1**		GR39	**1**			**1**	**1**	**1**
OR60		**1**	**1**	**2**			GR43	**2**		**1**	**2**	**2**	**1**
GR13/GR29 /GR46	**1**	**1**	**1**	**2**	**2**	**1**	GR44	**2**	**1**	**2**	**2**	**2**	**1**
GR4	**2**	**1**	**2**	**1**	**1**	**1**	GR45			**2**	**1**		**1**
GR18	**2**	**1**	**1**	**1**	**1**	**1**	GR49	**2**		**1**	**1**	**1**	**1**
GR19	**1**	**1**	**1**	**1**	**2**	**1**	GR50	**1**	**1**	**1**	**2**	**2**	
GR21		**1**		**1**	**1**	**1**	GR51	**1**	**1**	**1**	**1**	**1**	

Numbers in shaded boxes represent gene copies in each orthologous group of *Papilio* ORs or GRs. The blank space indicates the absence of orthologous genes in specific species. The remaining 45 OR and 26 GR groups that are not shown in this table are highly conserved across six *Papilio* species with 1:1:1:1:1:1 orthology. Pdar, *P. dardanus*; Pgla, *P. glaucus*; Pmac, *P. machaon*; Pmem, *P. memnon*; Ppol, *P. polytes* and Pxut, *P. xuthus*.

## Data Availability

All nucleotide sequences identified in this study have been deposited in the National Center of Biotechnology Information (NCBI) GenBank database, with accession numbers OP197207–OP197270 for *P. dardanus* ORs, OP197271–OP197328 for *P. glaucus* ORs, OP197329–OP197388 for *P. machaon* ORs, OP197389–OP197454 for *P. memnon* ORs, OP197455–OP197521 for *P. polytes* ORs, OP197522–OP197580 for *P. xuthus* ORs, OP197581–OP197637 for *P. dardanus* GRs, OP197638–OP197682 for *P. glaucus* GRs, OP197683–OP197736 for *P. machaon* GRs, OP197737–OP197796 for *P. memnon* GRs, OP197797–OP197857 for *P. polytes* GRs and OP197858–OP197906 for *P. xuthus* GRs.

## Supplementary Materials

The following supporting information can be downloaded at: https://www.mdpi.com/article/10.3390/insects13090779/s1, Table S1: Information on ORs and GRs in six *Papilio* species; Table S2: Primers used for the expression profiles of ORs and GRs in *P. xuthus*.
