# Supplementary material for: Genome-Wide Analysis of Odorant and Gustatory Receptors in Six Papilio Butterflies (Lepidoptera: Papilionidae)
Source: Insects. 2022 Aug 29;13(9):779. doi: 10.3390/insects13090779 (PMC9500883; doi:10.3390/insects13090779)
Supplement: Supplementary file 1 [file insects-13-00779-s001.zip › Table S2.pdf]

Table S2

Gene	Primer sequence (5'–3')	
	Forward primer	Reverse primer
**RT–PCR**		
OR1	GGTGGATCTTCATGACCCGTAT	CCAAAACTGCGCAGTGTCAC
OR2	CCGTAAGTTACATTTTACGGCG	CTTAGTGGCACTAATGCAAGCC
OR3	GTTATGTCAATTTGCGGTGCTAC	GCGTAAGACGCTAAAGACAGGTC
OR5	GGCATAGCAATAAAGGACTACGC	GGATGATAACGCGACAGCTTTAC
OR4	CCTACCTCGCAACGGTTCTAG	AGAATCCACCAGCTGACAACG
OR6	CATCCTGATGAAAATGACACACG	CGATCGTCATAACAATATGACCG
OR7	CACCGTACTACGAAGTGGCATT	CCGGAGCATTAAGTTGTAATGG
OR8	GGAGCCCATCCTAATTTACAGC	AGGGATCGAATCCCACATACAC
OR9	CAATTGTAAATACGCGGAGGC	AATGCCAGCAATTGATGGCT
OR10	CCGGTTTAGCGGAATTCATC	GATCGATACCAAAGGATCGAATC
OR11	ATTCGAAGAAACATCGTTGCAG	CGAAGGTGAGTTCTTCCGAAAT
OR12	ATACGGGACTTGGGGATTACTG	CACAATTGGCAGACAGTCTGCT
OR13	GGTTGAAGTTCTACGGAATGTGG	CAATAGCAGATGCGACAAAAGC
OR14	AGTACTTTTGATGGCGATCGG	ATGTCTCTTAATAGACTCCCGTCAGT
OR15	GACTAAAGCCTACCGCCAACTT	AATACAGGCACATTGTTGGTCC
OR16	ATGATTTGACGTATCCAGCAGG	GAACGGACTCGTCTATCCCAGT
OR17	AGGCTTGCTGAAACTGACGAAT	GACACCGAAATTCTCCCATCC
OR18	ATCCTGATCCTGACTGCACATG	ATAGAAATCGGGGCTACCGAT
OR19	ACATACAATACCGGGAGATGGAT	CAAAACTAACTCCGATTGTGACG
OR20	GCGTTGCATTTTGTCCTACTGT	GTCGTACTCATTTAACGCCGTC
OR21	AACTTGGTCAGAATTGCTGGGT	CACCGGCCTTGGATAAATTACT
OR22	GATGACGTCATGAGGAAGAACG	CATGGCCATCGAGAAAAACTC
OR23	CTGCAGAGATGATGGTGCTGTT	AATTCCGCTGATAGAACTTCCAC
OR24	CTGTGTGCAAAATGCTCATTCTC	CGACTAGCCAAACAATCAGCTCT
OR25	ATAAAAGGACCTGTGTTTGCACC	ATACAGATGATCCAGGCCATGAC
OR26	ACGGAATCCTTTCCACACATCT	AATTGACCAGCAAGGAGGATAAC
OR27	GCGAACCACCAGGTTATTACGT	CCGCTTTGAATACAAGCCTCTT
OR28	GCTACATCGCCTTCATCTCCTT	CTTAGACGAGTATCGGCAGGGT
OR29	AGCAATACTACCGGGTTTTGTTG	CATCCAATCAGAACTGTACGCTG
OR30	GAACCTTATTTATGCGCCTCG	CCAATAATTCCGTATTTCGGGT
OR31	AAGTTCGATTTTTGAACCGGTC	CAGCGGGCAAAGTAAATCTAAAC
OR32	CGCGCAGTAGTATTTGCGAC	AGGATGCTGAGCTGCGTCTT
OR33	CTACCGGTATGGGCTCCATT	ATAGCAACGCGACATACCCAT
OR34	GTGGTACTGGGGCGCATATAT	AACGGTTTCTGAGTCCGCAT
OR35	CTTTGCACCGATTTGCATTG	CAAAATTCCGTCCGACATTTG

OR36	GATGTTCCAAATGGTTGCTCAC	ATCTTAGCCATGGTCTGCACAC
OR37	GGTTCCTTGTCCAATTCCATTG	ATAAACATAAGACCCCGAACGC
OR38	GACGTTAATGACGAGGCGTACTT	AGATTGACCCATGGGTACGAGT
OR39	CACATTGATGGACACGATGTTTC	ACTGTATGTCGCTTCGACTTTTG
OR40	ATTCGATACTGGCCTTTCGCT	CTGCGATCTGGTAAGCACGAT
OR41	AAGGAAGTTGAACTTATGCTGCC	AACTTGCATGGTTTCTGAGCTCT
OR42	ATTTGGCTGAATAGTGACGTGC	AATGCCGTGATGGGATTAAGTT
OR43	CTGTCGGACAGTTGGTTCAAAT	CAGACAACAAGGACGTTGTGATC
OR44	ACAGTTGAAGTTTTGGCAGACG	CAATATTGCTACGCTTCCAACG
OR45	GCTATGGCTACCAGATGGGAAG	AGAGAGCGGAATGTAATGACCAG
OR46	ATGTGGATGCCTGTAAAACCG	CTATTGGTCTGCTCATACGCATC
OR47	ATCGCTGAAGTGAAGGAGGAAT	CAATTGGTAAGCAGTCGCACAT
OR48	GAAGCAATGCAATCGTGAGACT	AGACAATTGGTTCCCCTGGTAG
OR49	GCTTTTGAAATGTGGTTCCCTT	CTGTGATCGCCAGTTCCAATT
OR50	CGGTGGTGGTTGGATAACATC	AATTAGTGAATGGTCCAGCGG
OR51	CACAAGATACAAAGTGAGCCTGC	GTAGCGCTCTTTGACAACGAAT
OR52	ATGAATTGGCCGACATCTGG	GCTTACAGCCATAAACGGCTC
OR53	GCTGGAACTTTCGTTCTTTGG	ATTTGACCAGGCTTGAATGTTG
OR54	CGGGATATTTTTTCGCAACAC	CCGCTTTCATTATATACGGTGG
OR55	AACGCCTTGTTAACAGATGGTTC	AGCAAGAACAGCTGACCACACTT
OR56	GATTGTTGAAGCTTGGATGCC	CTGCGCTGAGACTCAATGGTT
OR57	CGAATGGGAGGACGTGATGT	GTGTGTCGCTCGTCATCCAG
OR58	GACAATGTTTCGATTGGTCGG	ATGGATCGAAGGGAAACCAGT
Orco	CAAGGCCTGGTATCCTTTCAAC	AATCCATCGATCTTCGTAGCCT
GR1	ACGACTTTGAACTGTGGCATTC	GTGCACTTCGATCTATTGCTCC
GR2	GAACAAGAACAACGTGACCTGC	AGAGGAAGCCGTCAAGTAGAGC
GR3	GATGGTTGACTTCAAGCTGGTG	ATTCATCCAGGAGAGTTCGACC
GR4	GGATATTTAGTCCGTGGACCGT	CAACTATCATTCCTTTCCCACG
GR5	CGCCTTTAAACGTGCTAGCTAT	AGTTGTTACCGCACCAAGTACG
GR6	AGCACAACAGGACATAGAAGCGT	AGCGTCGCTTGTTTTACATACG
GR7	CAATGCTTCGGTCTTAATCCTG	AACTCCACGTAAACGTCGACTG
GR8	GTGTTCTGCGTGAACCAATGAG	CCACAAATGGTACAGCTTTCCAT
GR9	CTTATCATCGCTTCAAAATGGC	ATAATCTTCCCGCAAATTACGC
GR10	GCTTATTAACTGGCGTTGCTGT	CGCCATTGATCGATGTGTAGAT
GR11	CTTAGTTGCAAGCTGATGGGTT	AGGCACTGGAATGGAAAATGAT
GR12	CAGCACAACCTGCATAACAATG	CCACTGATTACGTCGTCGACTC
GR13	GTAGTTCACGGTACGTTAGCGG	GTATTGTGAACGAGAATTGCGC
GR14	CAACGTACCAGAGCCATCTCTG	AACGACCACTGATGCCATAGAC
GR15	GAGACTTCGGGCAATCAGAATT	AGACTTCGGACTTCGGTCAATG
GR16	ATACCGGCACATTTAAGTCACG	ACTTCGGACCTCTGTCAGTGCT

GR17	CCTGTTTGCAATTGTGTTTCG	CAACTGCCATTTTATTGCAGC
GR18	AATTGCCCACCACATTGTTATC	CCGGGTAATTTATCCTGGAAAT
GR19	CGACAATAAAATCGCCTTCGA	TGACAAGGCGAGAAGAATGGA
GR20	CTTTTGCTACGGGTACAATTGC	ACACCAACAAGTTGAACGGAAG
GR21	GTTCCTTCGTTGCTGTTTCATC	GAGCAGTATGGTCCAAGAAGCTC
GR22	GGAATGCTTCGAAAACGTGTG	CTTGCACCCTCATACATTTTGG
GR23	AGATATCGTTTGGGCGAACG	GTCCAATAAAGTCGACGTCCAAT
GR24	GATCGACAGACGCTATTGACTTG	GACATCCACATTACGTTCCGAT
GR25	ATCGGCTTTTCTTACGCGAG	ATAAACGCCGTGGCCAGAG
GR26	GCAGCTTTGTCGCCGATATTA	AACACGCCCACGTTGAAAAC
GR27	GGTTTGTTGTGGTCACTACGCT	CAAGTATCGCCCATGTCTTTGT
GR28	CTTCAAGCCATCGATCGATATC	CCACTGCTTTAGACGTCCAAAG
GR29	AACATCATATATCTGGTGCGTGTG	AGCTTAGAGGTAATGTTGCGTCC
GR30	CATTTCCTCCCACTGTGTTAGC	ACGCTTGAACCATTGTATCCAG
GR31	ATTGGATCCATTCTGGACGAAG	CTTTAGCTGAATTTGTCTGGCG
GR32	CTGGGATGTAACACGCAAAACT	AAGCTGTGTACGTGGTCAGTACG
GR33	CACGTTTGTTACGAGGGATGAT	ACTCCTTGTGCTGATAACAGCTG
GR34	ATCGGATCAATTAGATTGCGTG	ACAGCATTACACAACTGGGAGG
GR35	CATTTTCGGAACAGTTGGAATG	AGAGCCGAAAGCTTGTATTCG
GR36	CTTTATGGTGGGATTTTCGCT	GCAACCGCATATCCACATCTAT
GR37	TCGTTTGTTGGAGATCAACCAC	TAATAACGCTGACAATGGTCCG
GR38	AAGGAGATCGTTTATGGAGGATG	GTTGAACCTCCATGTACCAACG
GR39	CGTTAATGTATCTCGCGTGACAT	ATTACGGTCATCGGAAAACTCAG
GR40	ATCGTGGCATGTGAATACGTGT	CATCCTTATGGCCCTTACTTCG
GR41	GATGTTATCGTCGTGTCGAGGTT	TACAATCGTGTTCGCACAATGAC
GR42	CCTATTTTGGGCAACTACATGG	GTTTAACATTGTCCCTTGCTGC
GR43	CAATGTGATCGTCTTCCTGTGC	ATCAGAGATTTTGAGCGCTTCC
GR44	CATAGCCACCAGTGCTGTTCTC	GTACGTGTGTGCAAGCTTTGCT
GR45	ATTCGTATGTGTGTGCGCTGTT	CGACACTCCGAAAATTTTGTTG
GR46	GTGCAGATGGGATGACAGTATTG	GCTCTGACGACGCAACAAAC
GR47	GTGCTTTGTACTTTGGCACAGTG	TGTTCACGGTTTTATATCGCTCC
GR48	AGTTTTCAAACTCAAACCGGC	GAGTCCTCTTCTAACCTGATTCGA
GR49	GGAAGGAAACTGAATCGTGGAT	ACAAATGCAAAGGTAAAGCTGC
RPS4	AAGTTGACGGCAAAGTGAGGAC	TCATCCTAGTGGCGAATGTGTG
**qPCR**		
PxutOR5	TGGACGAGACAGTAGTAAAG	CAAATGGCGATAGAGCTAAC
PxutOR14	GGAGTTATGTTGTTCCTGAC	ATCCATAGTGTGCCAGTTAC
PxutOR15	CCTTCAGAATGTTCAAGTGC	CAATCATAGGCAATTGTGCG
PxutOR16	GTGTGCGTTTAGCTATCTAC	GTCTGAACATAGAGCGAATG
PxutOR22	CATGGTCGTACAATTCTTCG	CCAGCATACACACCAAATAC

PxutOR23	GTTGCTGTGGAAGTTCTATC	CCATTCCGCTTCGTATATTG
PxutOR26	CTTACGATACCAGTGTGATG	CGCTCCTACTTCTTCATTAC
PxutOR27	CAGTAACGAAGTGACAGAAG	CCTCTTGCTCAATATCAGTC
PxutOR32	TATCGGATTCGTGACAGTTC	TTCATTCTGAGGATGCTGAG
PxutOR34	GGCAAGTATTAAGTGACCAC	CATGACGAGGTAACCATTAG
PxutOR35	GGCTGTTATACGGTTTGATG	AAGTCTTCTGGTTGGGTATC
PxutOR36	TGTAAGGAATCGTCGTACTC	GTTGTCTTCAGGATCTTAGC
PxutOR39	TCCAGATTACTTGGATTGCG	TCATAGGCTTGCGATAAAGG
PxutOR45	GATCTACATGGTGACAATGC	GAATGTAATGACCAGCCTTG
PxutOR47	GTGAAGGAGGAATTAGATGC	GCAGGAGTTTTAGACACATC
PxutOR52	CATTCTCTGTCGTCAATCTG	GGCGTACCAACATAATACAC
PxutOR56	GATGGTGACTATGGTTGATC	TCTTTCTGCATATCAGGAGG
PxutOR57	ACTTCGCCTACTTCTACAC	CTGCTTCGTCCAAAGATG
PxutOrco	CTGCTGGATGTAATGTTCTG	GATCTACCGTGTCTGGAATC
PxutGR1	ACCATAATTTCCCTCTACGG	GCGAACTTGGAACTCTAATC
PxutGR10	AGTATGGGTCTAACATCTCG	AGCAGAACTAATGGTCCTAG
PxutGR11	GATCACGACCAATACAGAAC	GGCAAGGTATGAAGTATGTC
PxutGR12	ATCCACGGAGATAAAGTAGC	GATCTTGCGGTGAACTATTG
PxutGR16	GATTGTTCGATGTGGCTATG	AATGATAGACTTCGGACCTC
PxutGR19	TACGGAGCAACCTACATAAG	TAAGTCGCAATATAGCCCAG
PxutGR21	GGCTTACGATTTCCTGAATG	CACAGTCATCGCAATACTAG
PxutGR31	GTTCTGATGCTATGTACTGG	CTATATTCTTGGTGGCTTCC
PxutGR38	TTCCTTATGGCATCATCAGG	CATTAGTGTTGCGTCCAATG
PxutGR44	CTCATTGAGGAGCCTATTTG	CTGCCATTATACAAGTGACC
PxutGR45	ACATCAAGAATACGGAGAGC	GAAAGAATGACATGACCACC
PxutGR46	TGGTGAGTAACTCTGTGATC	TCTCTGCCTCCATACTTAAC
PxutGR48	GACGTATCGCTGATATGATC	CCTGCACTAAATGTCTCTTC
RPL8	AACCTGGAAGAGAAGATGG	GCTTAACTCTAGTACGCTTG
